# Medical Gossip

**Published:** 1862-10

**Authors:** 


### Art. X.—MEDICAL GOSSIP.

The meeting of the British Medical Association held in London, under the presidency of Dr. Burrows, in the course of the past quarter, gave excellent promise of increased and increasing prosperity for the Association. The cordial reception and welcome of the College of Physicians, and almost portentous hospitality of the College of Surgeons, must have been most gratifying to the members of the Association, as it was honourable to the two corporations. Whether the meeting be regarded from the fact of its being the first held by the Association in the metropolis, or it be judged by the value of the addresses delivered by the President, Dr. Walshe, Mr. Paget, and Dr. Sharpey, and the papers submitted to it ; or it be considered from the less patent, perhaps, but not less important indications of professional brotherhood engendered by the genial gossip and friendly intercourse which characterized the intervals of relaxation from graver duties, the meeting will always be looked upon as constituting an epoch in the history of the Association—one, we trust, to be marked by renewed vigour in its operations, and more widely extended professional benefit arising from them.

A curious and noteworthy illustration of the quarantine system pursued on our coast occurred in the Thames in the course of August. An iron screw-steamer, called the *Melita*, of about 800 tons, left Nassau on the 2nd of August, having, it is said, previously run the blockade at Charleston. She had on board a crew of some fifty officers and men, and twenty-three passengers, with a general cargo, including about twenty-five bales of cotton. On the voyage home, which lasted barely twenty days, no fewer than seven of the passengers and the engineer of the vessel died, of yellow fever, it was said, and were buried at sea. The captain and two of the passengers landed at Dover, leaving the ship in charge of the chief mate and a pilot. She then steamed up the Thames, evaded the quarantine regulations at Gravesend, and arrived in the Victoria docks. There she was boarded and overhauled in the usual manner by an examining officer of Customs, who in the course of his search heard from some of the crew in an incidental way, what had happened on the voyage, and which he deemed it his duty to report immediately to head-quarters. He and two or three of his assistants, with a pilot and some other people who had gone on board, apprehending nothing wrong, were condemned to remain there, as were also all the surviving passengers and crew, during three or four days and nights. The vessel was pushed off into the basin, detached from the quay and away from the rest of the ships, while a vigilant guard was kept over her lest any one on board should escape, and, what was much less likely, lest any one should approach her. A man detected in the act of attempting to take a bottle of beer or spirits on board, was put under arrest for the greater part of a day. Another who had gone on board before the discovery was made, threw a written message on shore to explain his absence to his family, but it was seen and kicked into the basin. It is, no matter of surprise that the vessel should have eluded the quarantine watch at Gravesend. Ordinarily, when a vessel arriving from a foreign port is challenged by an officer of Customs at Gravesend, the captain is asked a series of set questions as to the health of the crew and passengers—whether there had been any deaths on board, and the like. That is said to be the invariable rule with respect to sailing vessels, but it would appear to be somewhat relaxed in the case of steamers when under full steam, the officers in charge of which are usually impatient of being stopped in their course to reply to all the prescribed interrogatories, especially when they are themselves satisfied with the sanitary condition of the vessel. In such a case, the customary questions are condensed into the laconic one of "All well?" which in this case was answered in the affirmative, and the steamer sped on her way. Where deaths have occurred on board a ship from any contagious disease, and the attendant circumstances justify such a course, she is detained at Gravesend until the case can be reported to the Commissioners of Customs. The result usually is an order in Council, on the authority of which the vessel is sent to lie off the Motherbank during the appointed interval of quarantine; but in this instance the *Melita*, which having run the blockade at Charleston, ran also, so to speak, the quarantine blockade at Gravesend, had been put upon quarantine in a dock crowded with shipping and in the immediate vicinity of a populous suburb of the metropolis, under the peculiar circumstances that have been related. When overhauled by the Custom-house examiners in the docks, she was found to be remarkably clean, and the captain is said to have taken every precaution to prevent the spread of infection. The owners, too, showed laudable zeal to carry out the orders of the Custom-house authorities. After three days' quarantine, the vessel was declared to be free from danger, and the passengers and crew were permitted to leave her. By a peremptory order of the Commissioners of Customs, every conceivable article of wearing apparel and bedding which had belonged to or been used by the persons who died was collected together, and burnt in the furnaces of the vessel, under the superintendence of a surveyor of Customs. At his discretion, money and articles of jewellery, some papers of moment to relatives, including in some cases bills and the like for considerable sums of money, were exempted from the order for destruction.

From the history of this case we learn that the *Melita* had a narrow escape from the fate which befell the ill-fated *Eclair*. By the wisdom of the quarantine authorities, when that vessel reached these shores with yellow fever on board, the sick and well were cooped up in her, and, as a consequence, many valuable lives were needlessly lost to the country. The same course was adopted in the case of the *Melita*, and the same results would unquestionably have followed had it not proved that there was no yellow fever on board at the time. Whether the deaths on board resulted from this disease or not is doubtful, but it is high time that our quarantine system was dominated by some wiser regulations than those now in force. As it is now practised, the system appears to be calculated to excite the greatest amount of public alarm, with the least quantity of public good. It would be well if the wise suggestions of the Quarantine Committee of the National Association for the Promotion of Social Science were carried into effect by the Legislature.

Lunacy troubles have cropped out in Australia, if we may judge from the report of a trial for libel, "Bowie against Wilson," in the law courts at Melbourne, the plaintiff being the surgeon-superintendent of the lunatic asylum called the Yarra Bend, the defendant one of the proprietors of the *Argus*. That paper in September last contained some severe strictures on the management of the asylum. These animadversions were in a great measure based upon evidence given before a Committee of the Assembly, on the reports of inquests, and other sources of information. Some, however, were stated to be "on rumours," and the whole were pointedly directed against the surgeon-superintendent in proof of his inefficiency. He brought his action for libel, and the defendant justified all the material statements. The result, after nine days' investigation, was substantially a verdict for the defendant, the plaintiff recovering on one issue only, with 100*l.* damages. The evidence showed that the plaintiff is a practitioner of the old school, who retains the exploded theory that restraint is indispensable, and he has continued to carry this into practice to an extent universally condemned by modern practitioners. Considerable excuse may be conceded to him from the fact that the increasing number of patients from year to year has always kept in advance of the means furnished to him by the Legislature for better treatment. To some extent he is made the scapegoat for the inefficient state of the institution; but against this it appears that he not only did not remonstrate or "make requisitions," but pertinaciously maintained the sufficiency of the establishment against the opinions of other competent authorities. In short, he trusted to harsh restraints in lieu of watchful attendants. The result of the complete exposure of abuses effected by this trial has been to direct public attention to the state of the institution, and it can hardly be doubted that early next session the legislature will make more ample provision for the custody and treatment of lunatics than it has hitherto been induced to do. It is understood that Dr. Bowie is to be allowed to retire upon an allowance of half his present salary, and the Government will probably endeavour to obtain an experienced surgeon from England. Though we may lament that the result of this trial is more disastrous to the unsuccessful plaintiff than he deserves—though, while he is clearly not free from merited censure, he is made to answer for some things for which the Legislature is to blame,—yet it cannot be doubted that great benefit will flow from the searching investigation which the *Argus* has been the means of bringing about—an investigation infinitely more complete than an inquiry before a parliamentary committee, whose long and diluted reports stand for nothing against the pithy conclusion of the jury on a plea of justification—"We find for the defendant." The dissemination of the evidence by means of the press affords an amount of instruction on the management of the lunatic asylum which has never before been afforded, "and every man ought now to feel," says our report, "that as an elector, he too has a duty to perform which it will be criminal to neglect." A rule *nisi* has been applied for by the plaintiff in this case, on the ground that the chief justice did not put the separate issues to the jury, and was granted by the court.

The disastrous news from the Federal armies before Washington form a sad comment upon the noble efforts of the United States Sanitary Commission. The history of this commission will be found on another page, and it will be observed that the forebodings expressed by the secretary of the commission, after a preliminary inspection of several of the camps, have been but too fully realized. The gigantic force in the field has been an insuperable obstacle, it is to be presumed, to an efficient inoculation of officers and men, during active campaigning, with the admirable lessons taught by the Commission; and the political state of affairs has doubtless induced that apparent apathy, neglect, or improvidence of Government departments, which is to be inferred from the latest letters from the forces engaged on the Rappahannock and at the second battle of Bull Run. It would almost seem as if the report of the Sanitary Commission upon the cause of the loss of the first battle of Bull Run, and which we have quoted elsewhere, would in all essential points apply to the second.

We cite the following statements, made by the Baltimore correspondent of the *Times*. They form a painful but instructive comment upon our article on the Sanitary Commission:—

"General Pope's despatch, falsely calling the battle of Groveton, on Friday, August 20, a victory, was warmly hailed in Washington, and preparations were at once made to show solicitude for the wounded. A train of 200 ambulances was sent forth, containing many Government *employés* and private citizens, who long will remember their experiences on that eventful trip. Shortly after they had started, the disastrous news of Saturday's battle reached Washington, but the ambulances were beyond recal. They travelled all Saturday night, and arrived on Sunday near the field of battle. They found it in the hands of the Confederates. A few ambulances went boldly forward, and fell at once into the hands of the Southern pickets. Such of the wounded as were able to drag themselves towards the ambulances were accommodated on them, and the Government *employés* and civilians repaired to the nearest station with a view to returning by rail. Here they were peremptorily refused admission, and all the accommodation reserved for wounded soldiers. Weary, footsore, half-starved, out for two nights without sleep, these good Samaritans dragged themselves back on foot to Washington, heartily enraged at their treatment by the Government. It was of a piece with its action in all other matters. Hundreds of surgeons have been telegraphed for from Northern cities. They repaired to Washington, where no accommodation, no orders, no patients awaited them. Many of them returned in high irritation to their homes.

"One of the most heartrending thoughts, in connexion with all these recent battles, is that, comparatively, a very small number of the wounded has hitherto been brought into Washington. That the number of wounded in these battles is enormous seems generally believed. Humanity shudders and recoils at the thought of thousands of frail, suffering, anguished bodies, many of them famished when they received their wound, lying for forty-eight or seventy-two hours without food or water or kindly care—many a poor weary soul escaping from its racked and fevered casket from sheer want of human tenderness and sympathy.* But, though there are thousands of sufferers left behind, Washington has this week seen a sight which might well draw tears from a stone, but which seems scarcely to have ruffled the serene indifference of the stolid American nature. On Monday a long train of maimed and bleeding and mangled wretches, most of them wounded the Friday before, approached over the Long-bridge. No one was there to look after them, and there for hours lay this mass of tortured and writhing humanity, while pools of blood trickled from the carriages and left a ghastly trail behind. Many a poor fellow not deprived of the use of his legs, untended in the churlish and bewildered city, wandered to the railroad station, with heart fondly turning to some far-off home, possibly among the hills of what was once happy and peaceful New England. He who could witness that sight and not from his heart abhor the grasping vaulting nature of those politicians who, rather than accept what has long been inevitable, would month after month subject poor human nature to such unutterable anguish, must have been cold indeed. But no sign of emotion did Washington deign to give or betray. The same idle *poco-curante* crowd at Willard's Hotel, the same knot of sharks and contractors fattening on a nation's woes, the same frail and miserable women profaning with repulsive levity streets and buildings which the Angel of Death had marked for his own,—well may the bewildered stranger ask himself with sickening horror, Is this a Christian nation which conceives the best tenants for its churches to be the maimed and agonized and dying, and can look at such sights undaunted and unmoved? Can it bear the thought that thousands of its sons should lie for days starving and with wounds festering on the field of battle, finding no relief till mortification, more merciful than Christian man, preludes a final and painless repose?"

Again:—

"Nothing is more conspicuous throughout these memorable months of strife than the superiority, in all qualities essential to the formation of armies, of the Southern section. Their officers are not boon com-

---

* "The wounded lay days—long days and long nights—some of them a week of long days and long nights—among these putrid corpses, wanting care for their wounds, wanting food, wanting water, calling in faint voices to occasional passers by, friend or foe, for help, and receiving none. These are facts, disgraceful as they are stubborn. . . . Although our authorities must have known that the dead still remained on the field, an entire week passed before adequate means were taken to hide in the earth the revolting spectacle."—*Corr. New York Tribune.*

panions picked out of some bar-room and promoted to the distinction of wearing shoulder-straps by reason of skill and dexterity in conducting municipal elections, or, in the language of this country, wire-pulling. The Southern officers are, and have always been, gentlemen, and their followers have been accustomed all their lives to regard them as belonging to a superior class; and, therefore, discipline and subordination are natural and easy. I am very far from saying that in the Federal Army there are not also many gentlemen who are officers; but it is impossible not to perceive that there are also many who are not fit for command. Whatever may be General M'Clellan's military qualities, no one can be for one moment in his company without perceiving that he is emphatically a high-bred, modest, unassuming, and courteous gentleman; and the same may be said of hundreds of others whose names will suggest themselves at once to Englishmen familiar with this country. But there is a lower deep, in which are to be found officers of Volunteers whose democratic antecedents have always placed them on an equality with their men, and who are now called upon to introduce another system of government among reluctant and recalcitrant followers. Discipline and regimental organization are necessarily aristocratic. In this more than any other point the North has broken down. It was thought that the long organization and drill enforced last winter by General M'Clellan would wean his men to habits of discipline. So it might have been if success had accompanied their advance on Richmond. But the world has seldom seen such demoralization as accompanied their retreat from the James River to Alexandria."

A correspondent of one of the American papers also writes of the Federal officers:—" In my last I alluded to the great number of officers that had business in Alexandria last Saturday and Sunday while the fighting was going on in front. From the books at the Market-house and City Hotel alone, I learn, that on Sunday last 155 officers registered their names for dinner. Recollect this is only the number at two hotels, while there are hundreds of smaller hotels and boarding-houses that were besieged by them. The number of soldiers that straggled into and were scattered around the city last Sunday, could not have been less than 6000 or 8000. To-day only seven officers registered their names at the two hotels above-mentioned."

This letter is dated the 8th September.

Finally, General Pope tells us, in a despatch of 3rd September, that he was compelled to retreat behind Bull Run, if he "wished to save men and animals from starvation." The horses had been two days without forage, the men were exhausted from fatigue, and were very short of provisions, and his urgent requests for forage and rations had been unattended to.

